# Supplementary material for: A Metabolomic Profile Predictive of New Osteoporosis or Sarcopenia Development
Source: Metabolites. 2021 Apr 28;11(5):278. doi: 10.3390/metabo11050278 (PMC8145554; doi:10.3390/metabo11050278)
Supplement: Supplementary file 1 [file metabolites-11-00278-s001.zip › metabolites-1191112-supplementary.pdf]

Supplementary Table 1. Detected metabolites.

Pyruvate
Butyrate
Lactate
2-Hydroxyisobutyrate
2-Hydroxybutyrate
3-Hydroxybutyrate
Fumarate
2-Oxoisopentanoate
fourOxopentanoate
Succinate
Isethionate
5-Oxoproline
Citraconate
4-Methyl-2-oxopentanoate
Glutarate
Malate
Threonate
Octanoate
2-Oxoglutarate
2-Hydroxyglutarate
Pelargonate
Urate
Glycerophosphate
cis-Aconitate
Isocitrate
Citrate
Glucuronate
Dodecanoate
NAcetylaspartate
Hippurate

Azelate
Gluconate
Mucate
3-Indoxyl sulfate
Urea
Gly
Trimethylamine N-oxide
beta-Ala
Ala
3-Aminoisobutyrate
2AB
N,N-Dimethylglycine
Choline
Ser
Diethanolamine
Creatinine
Pro
Val
Betaine
Thr
Taurine
Pipecolate
Hydroxyproline
Creatine
Ile
Leu
GlyGly
Asn
Ornithine
Asp
Hypoxanthine
Proline betaine

gammaButyrobetaine
Gln
Lys
Glu
Met
His
Carnitine
Phe
3-Methylhistidine
Arg
Citrulline
Tyr
o-Acetylcarnitine
Trp
Cystine
Uridine
Glycerophosphorylcholine
PhePhe
